# Normal‐Appearing White Matter Injury Mediates Chronic Deep Venous Hypoxia and Disease Progression in Multiple Sclerosis

**DOI:** 10.1002/acn3.70354

**Published:** 2026-02-27

**Authors:** Xinli Wang, Huiying Wang, Zhizheng Zhuo, Ai Guo, Ke Lv, Decai Tian, Chao Chai, Yunyun Duan, Shuang Xia

**Affiliations:** ^1^ Department of Radiology First Central Hospital of Tianjin Medical University Tianjin China; ^2^ Department of Radiology Tianjin Medical University General Hospital Tianjin China; ^3^ Department of Radiology, Tianjin Institute of Imaging Medicine Tianjin First Central Hospital, School of Medicine, Nankai University Tianjin China; ^4^ Department of Radiology Beijing Tiantan Hospital, Capital Medical University Beijing China; ^5^ Department of Neurology Beijing Tiantan Hospital, Capital Medical University Beijing China; ^6^ School of Medicine Nankai University Tianjin China

**Keywords:** cerebral venous oxygen saturation, microstructural alterations, multiple sclerosis, quantitative susceptibility mapping, white matter

## Abstract

**Objective:**

To explore how cerebral hypoxia and Normal‐Appearing White Matter (NAWM) integrity affect MS lesion burden and clinical course.

**Methods:**

Seventy‐nine MS patients, including 13 clinically isolated syndrome (CIS) patients and 66 relapsing–remitting multiple sclerosis (RRMS) patients, and 44 healthy controls (HCs) were recruited from CLUE, NCT04106830. Quantitative susceptibility mapping (QSM) was employed to evaluate the changes of cerebral venous oxygen saturation (SvO_2_) in deep cerebral veins. Diffusion tensor imaging (DTI) and neurite orientation dispersion and density imaging analyzes (NODDI) were employed to evaluate microstructural alterations in deep brain white matter (WM), including WM lesion and NAWM between MS and HCs. Partial correlations analyzes were conducted to examine associations between imaging biomarkers and clinical indicators. Mediation analysis was used to evaluate the relationship among SvO_2_, microstructural alterations, lesion volumes, and clinical indicators.

**Results:**

Compared with HCs, patients with MS showed significantly decreased SvO_2_ in the internal cerebral vein (76.56% ± 1.34% vs. 78.80% ± 0.86%, *p* < 0.001). Advanced diffusion metrics revealed extensive microstructural disruption in both WM lesions and NAWM (NAWM mean diffusivity [MD]: 1.03 ± 0.12 vs. 0.90 ± 0.04 [×10^−3^ mm^2^/s], *p* < 0.001). Furthermore, microstructural disruption of NAWM (MD and orientation dispersion index [ODI]) significantly correlated with SvO_2_ of the ICV (MD: *r* = −0.307, *p* = 0.036; ODI: *r* = −0.279, *p* = 0.036). Critically, mediation analysis demonstrated that deep brain WM hypoxia (ICV SvO_2_) associated with greater lesion burden and clinical disability via NAWM damage as an intermediate pathway.

**Interpretation:**

In MS patients, lower cerebral SvO_2_ (compared with HCs) is statistically associated with microstructural alterations in the NAWM. Our mediation models are consistent with a pathway whereby lower SvO_2_ is associated with greater lesion burden and worse functional scores via its association with NAWM damage. These findings support the exploratory value of SvO_2_ and NAWM integrity as potential biomarkers for monitoring MS progression, which warrants validation in further longitudinal studies.

## 1 Introduction

Multiple sclerosis (MS), the most common neurological disorder affecting young to middle‐aged adults, often progresses to cause physical disability and cognitive impairment [1]. While earlier studies primarily emphasized the immune system as the initiator of inflammatory demyelinating, recent research has proposed cerebral hypoxia as an alternative potential mechanism [2]. Type III MS lesions exhibit a high induction of hypoxia‐inducible factor (HIF)‐1α, suggesting that hypoxia may serve as a potential early trigger of white matter (WM) inflammation [3]. Some studies have indicated that demyelinating lesions in MS patients experience both virtual hypoxia (metabolic or histotoxic) and real hypoxia (oxygen deficiency) [4]. Despite these advances, the spatiotemporal dynamics linking chronic hypoxia, neurodegeneration, and lesion evolution remain poorly mapped. Critically, the relationship between cerebral hypoxia and the neurodegenerative cascade requires systematic investigation.

Ge et al. reported significantly reduced visibility of periventricular WM venous vasculature in MS patients compared with healthy controls (HCs) [5]. Zeng et al. similarly demonstrated that patients with relapsing–remitting MS (RRMS) had lower mean scores for internal cerebral veins (ICVs) and their main tributaries than HCs [6]. Nevertheless, these studies mainly investigated venous morphology rather than cerebral oxygen metabolism. Cerebral venous oxygen saturation (SvO_2_) is a critical indicator of cerebral oxygen metabolism, oxygen extraction, and neural activity. A previous study employing quantitative susceptibility mapping (QSM) has explored SvO_2_ alterations in various neurological conditions, including acute ischemic stroke, traumatic brain injury, hemodialysis patients, as well as in healthy subjects [7, 8, 9, 10]. However, regional SvO_2_ (rSvO_2_) in major cerebral veins has not yet been thoroughly investigated in patients with MS.

The deep brain WM, including periventricular and deep WM regions, is disproportionately affected in several neurological disorders, such as small vessel disease (SVD), MS, certain leucodystrophies, and diabetes [11]. John Dawson characterized MS lesions as plaques primarily centered around small periventricular veins, most prominently located in brain deep WM. This region is notably under‐vascularized, making the local oxygen supply particularly vulnerable. Besides visible lesions, this area also contains normal‐appearing white matter (NAWM). Models of diffusion‐weighted MRI allow detection of microstructural abnormalities occurring in MS patients, especially within NAWM, overcoming the limitations of conventional MRI methods [12]. Microstructural studies have confirmed ongoing demyelination and inflammation within lesions and NAWM, along with neurite loss, consistent with histopathological findings and data from other imaging modalities [13, 14, 15, 16]. Since studies on NAWM alterations in MS patients provide accumulating evidence for the disconnection syndrome hypothesis, potentially explaining the neural mechanisms behind their characteristic cognitive and motor impairments [17], and NAWM microstructural damage is associated with WM lesion progression, we not only monitor changes in lesions but also focus on alterations in NAWM.

Based on these considerations, we hypothesized that reduced rSvO_2_ in the deep cerebral draining veins may be associated with microstructural damage in the NAWM of the deep brain, which in turn mediates lesion burden and clinical status in patients with multiple sclerosis. Therefore, our specific aims were: (1) to assess variations in SvO_2_ within the draining veins of the deep brain WM; (2) to quantify the microstructural damage within deep brain WM subdivided into WM lesions and NAWM; and (3) to determine whether SvO_2_ and microstructural alterations in NAWM correlate with lesion volumes and clinical outcomes in MS patients.

## 2 Materials and Methods

### 2.1 Subjects

The study was approved by the local ethics committee (KY‐2019‐050‐02), and informed consent was obtained from all participants. This study enrolled 79 patients from a prospective, single‐center neuroinflammatory disease cohort at Beijing Tiantan Hospital, Capital Medical University (CLUE, NCT04106830) from January 2019 to May 2021. Clinically isolated syndrome (CIS) (*n* = 13) and RRMS (*n* = 66) patients were included in this study according to the following criteria. Inclusion criteria for patients were as follows: (1) diagnosis of clinically isolated syndrome (CIS) or relapsing–remitting MS (RRMS) according to the 2017 McDonald criteria [18]; (2) being in a clinically stable phase, defined as relapse‐free and free of corticosteroid treatment for at least 1 month prior to study enrollment and MRI acquisition; (3) presence of intracranial lesions; (4) Disease‐modifying treatments (DMTs) or immunomodulatory therapies were required during the remission stage for RRMS patients. Exclusion criteria for all participants (both patients and HCs) included: (1) history of other major neurological or psychiatric disorders; (2) presence of cerebrovascular risk factors, including a clinical history of hypertension, diabetes mellitus, hyperlipidemia, atrial fibrillation, or prior stroke/transient ischemic attack, which were assessed through patient self‐report and review of medical records; (3) patients receiving stable DMTs for ≥ 6 months; (4) any contraindication to MRI. Both CIS and RRMS patients were included to investigate the pathophysiological mechanisms of cerebral hypoxia and NAWM injury across the early spectrum of MS. We hypothesized that these processes are relevant from the earliest clinical stages of the disease. Additionally, 44 age‐ and sex‐matched healthy controls (HCs) without neurological disorders or visible white matter hyperintensities on MRI were recruited. Demographic and clinical information, including age, gender, disease duration, cerebrospinal fluid oligoclonal bands (OCB), the Expanded Disability Status Scale (EDSS) were collected. The patient's HCT values were obtained from routine blood tests conducted after hospital admission, with the time difference between testing and MRI scanning being less than 3 days. The HCT reference values for healthy control subjects were established based on measurements taken around the time of the MRI scan at the Health Management Center. MS patients and HCs underwent comprehensive neuropsychological tests within 1 week of MRI scanning by trained neuropsychologists. Common cognitive function assessments by Mini‐Mental State Examination (MMSE), Montreal Cognitive Assessment (MoCA). Advanced cognitive function was assessed using the California Verbal Learning Test‐Second Edition (CVLT‐II), Brief Visuospatial Memory Test‐Revised (BVMT‐R), Symbol Digit Modalities Test (SDMT), and Controlled Oral Word Association Test (COWAT).

### 2.2 Image Acquisition

MRI scans were acquired using a 3.0‐Tesla Philips scanner (Philips Ingenia CX, Best, The Netherlands). Three‐dimensional (3D) T1‐weighted images were used for brain volume measurements, 3D‐T2‐fluid‐attenuated inversion recovery (FLAIR) images for lesion measurement, QSM for cerebral rSvO_2_ measurement, and multi‐shell diffusion imaging for assessing WM microstructural integrity. The MRI protocol included: (1) Sagittal 3D T1 images, repetition time (TR)/echo time (TE)/inversion time (TI) = 7/3/880 ms, flip angle = 8°, voxel size = 1 mm × 1 mm × 1 mm, matrix size = 256 × 256, number of slices = 196; (2) Sagittal fluid attenuation inversion recovery (FLAIR), TR/TE/TI = 4800/340/1650 ms, flip angle = 90°, voxel size = 1 mm × 1 mm × 1 mm, matrix size = 228 × 228, number of slices = 165; (3) 3D flow‐compensated QSM images were acquired using gradient echo (GRE): TR/TE = 40.0/4.5 ms, flip angle = 20°, image resolution = 0.75 mm × 0.75 mm × 0.75 mm, number of slices = 200, echo space = 4.5 ms, echos = 8; (4) Multi‐shell diffusion imaging was as follows: axial 2D spin‐echo echo planar imaging (SE‐EPI) acquisition; TR/TE = 4000/88 ms; Flip angle = 90°; in‐plane acquisition voxel size = 2.5 mm × 2.5 mm; acquisition matrix size = 96 × 96; slice thickness = 2.5 mm; number of slices = 60; *b* values = 0, 1000, 2000 s/mm^2^, with 48 motion sensitive gradient directions for each non‐zero b values; SENSE factor = 2; multi‐band factor = 3.

### 2.3 Lesion Volume Analysis

Lesions were identified using the automated lesion prediction algorithm (LPA) from the Lesion Segmentation Tool (LST, version 3.0.0) within the Statistical Parametric Mapping software (SPM12, r7771) based on FLAIR image analysis. The output of the LST segmentation underwent manual quality checks, and lesion maps were registered to MNI space using 3D‐T1WI. Additionally, the volume of deep brain WM lesions was calculated.

### 2.4 Segmentation of Deep Brain WM and NAWM

**FIGURE 1:**
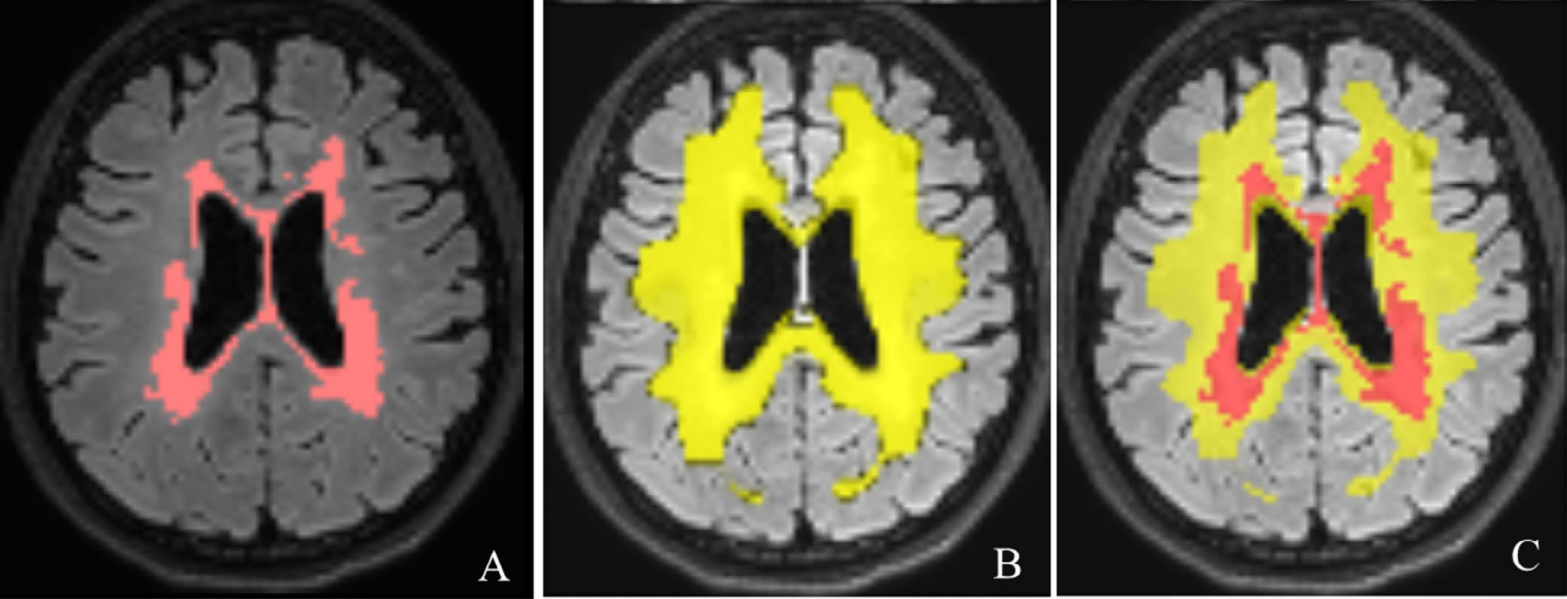
Segmentation pipeline in a representative multiple sclerosis patient. (A) FLAIR image showing white matter lesions (highlighted in red). (B) The deep brain white matter mask (in yellow), encompassing periventricular and deep white matter regions. (C) The normal‐appearing white matter (NAWM) mask (in pale yellow), derived by subtracting the lesion mask from the deep brain white matter mask.

The WM was segmented from the cat_wmh map in Computational Anatomy Toolbox (CAT12, version r1890) [19] to create a deep brain WM mask. This mask included deep WM and periventricular WM, aligning closely with the deep venous drainage area. To obtain the NAWM area, the lesion area was subtracted from the deep brain WM mask (Figure 1).

### 2.5 Image Processing for Susceptibility Quantification

**FIGURE 2:**
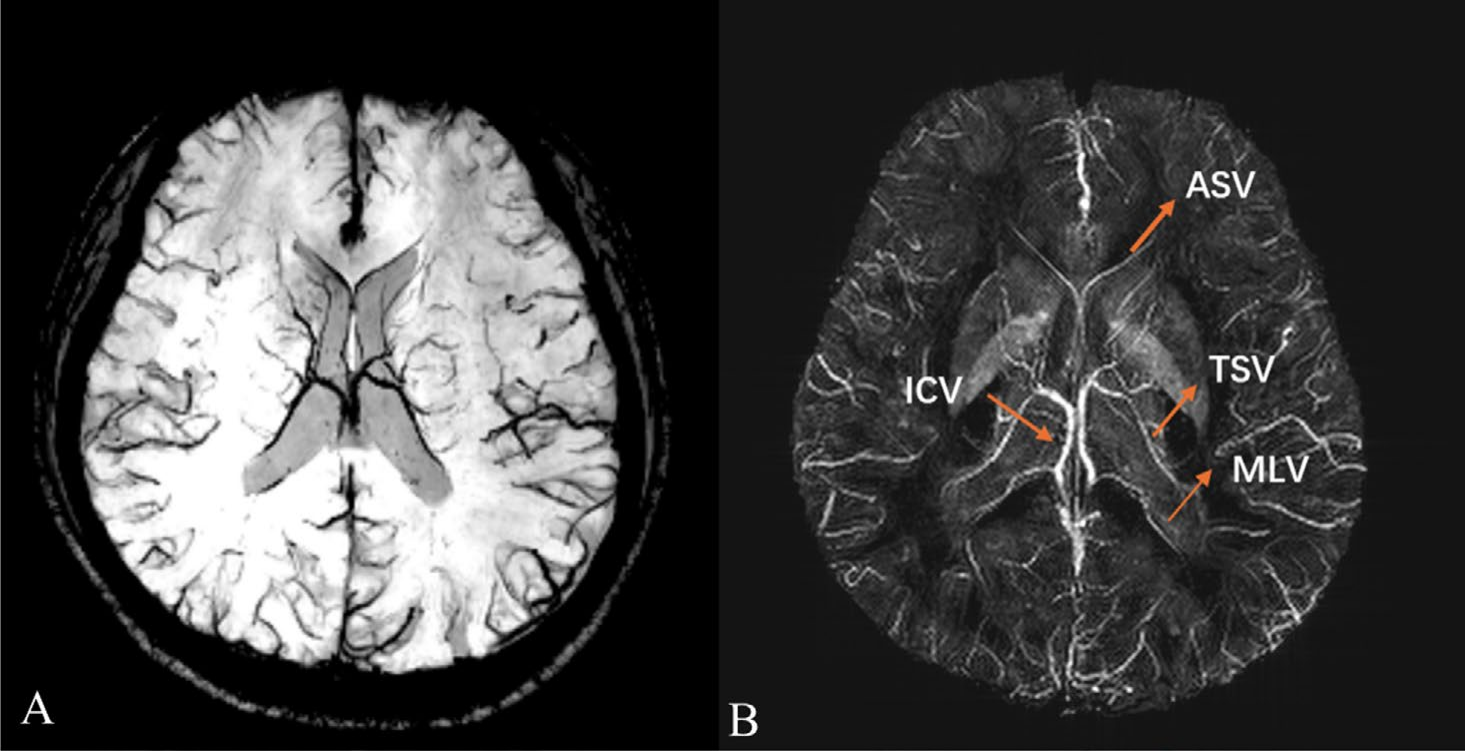
Deep cerebral veins in a representative subject. (A) Susceptibility‐weighted image (SWI) and (B) quantitative susceptibility mapping (QSM) clearly depict the deep cerebral veins, including the anterior septal vein (ASV), thalamostriate vein (TSV), medial‐lateral ventricle vein (MLV), and internal cerebral vein (ICV).

QSM images were reconstructed using the STI Suite MATLAB toolbox. Briefly, the reconstruction consisted of Laplacian unwrapping, background field removal, and dipole inversion by using the total generalized variation regularization. Magnetic susceptibility maps were reconstructed from raw k‐space data. Signal Processing In NMRI (SPIN) software (SpinTech Inc) was used to view the QSM data and measure the susceptibility. The susceptibility of deep cerebral veins in both hemispheres was measured from a maximum intensity projection (MIP) image. Maximum‐intensity‐projection (MIP) images were generated by projecting the 3‐D QSM volume along the axial direction (slice direction) using the MIP function in SPIN (SpinTech Inc.), with a 16‐mm slab thickness and 8‐mm step size to preserve spatial resolution while enhancing vessel conspicuity. A threshold of 70 parts per billion (ppb) was applied to depict the cerebral deep vein from background brain tissue. The cerebral deep veins, including the anterior septal vein (ASV), thalamostriate vein (TSV), medial‐lateral ventricle vein (MLV), and internal cerebral vein (ICV) (Figure 2A,B), were outlined in each slice by the two neuroradiologists; the intraobservers intraclass correlation coefficient (ICC) was 0.87 for average susceptibility and pixel. The average susceptibility and pixel of each vein were obtained for further analysis.

The QSM data provided a method to assess cerebral rSvO2 using the difference in susceptibility (Δ*χ*) between tissues. The SI units of susceptibility are quoted in ppb. The relationship between Δ*χ* and SvO_2_ is given by

(1) $$\mathrm{Sv}O_{2}=1-\left(∆\chi -∆\chi_{\mathrm{oxy}}\cdot \mathrm{Hct}\right)/\left(∆\chi_{\mathrm{do}}\cdot \mathrm{Hct}\right)$$

where $∆\chi_{\mathrm{oxy}}$ is the susceptibility difference between fully oxygenated blood and water (−0.03 × 4π × 10^3^ ppb), $∆\chi_{\mathrm{do}}$ is the susceptibility difference between fully oxygenated and deoxygenated blood (0.27 × 4π × 10^3^ ppb), and Hct is the average hematocrit of each patient and HCs.

Due to the finite spatial resolution of the QSM acquisition, partial volume effects may influence the susceptibility measurements, particularly in smaller veins or at the boundaries between veins and surrounding tissues. To mitigate this, we applied a threshold of 70 ppb to enhance vessel conspicuity and minimize contamination from adjacent brain tissue. Additionally, the use of maximum intensity projection (MIP) with a 16‐mm slab thickness and 8‐mm step size helped to preserve spatial resolution while improving vessel visualization. Nevertheless, partial volume effects remain a potential limitation in the accurate quantification of venous susceptibility and SvO_2_, especially in veins with diameters approaching the voxel size.

### 2.6 Diffusion Data Analysis

Multi‐shell diffusion images were preprocessed using a collaborative resource platform named Diffusion‐MRI (https://github.com/RDadarwal/Diffusion‐MRI), which incorporates DIPY (https://dipy.org/), Nipype (https://nipype.readthedocs.io/en/latest/), FSL (https://fsl.fmrib.ox.ac.uk/fsl/fslwiki/FSL), AMICO (https://github.com/daducci/AMICO), and MRtrix3 (https://www.mrtrix.org/). Image preprocessing included (1) image denoising using DIPY, (2) b0 images extraction and skull stripping using FSL, (3) eddy current correction using FSL. The diffusion tensor model was reconstructed using FSL, and NODDI model was fitted using AMICO. The NODDI model was fitted using the AMICO framework to derive maps of the intracellular volume fraction (ICVF) and orientation dispersion index (ODI) [20]. The standard three‐compartment NODDI model was employed, which includes: An intra‐cellular compartment (modeled as sticks with a Watson distribution for orientation dispersion). An extra‐cellular compartment (modeled as hindered diffusion). A free water (CSF) compartment (modeled as isotropic diffusion). In total, 4 diffusion metrics were calculated, including fractional anisotropy (FA), mean diffusivity (MD), intracellular volume fraction (ICVF), orientation dispersion index (ODI).

### 2.7 Statistical Analysis

All statistical analyzes were conducted using SPSS software (version 22, IBM). The normality of continuous variables was assessed using the Kolmogorov–Smirnov test. Continuous data were presented as mean (SD), and ranked data were presented as median (IQR). The Kruskal‐Wallis test, or two‐sample *t*‐test, were applied as appropriate based on data normality. The chi‐squared test was used to compare gender differences between two groups. Normally distributed continuous variables were analyzed using analysis of covariance (ANCOVA), with adjustments for age, gender, and total intracranial volumes (TIV). Partial correlations were conducted to estimate the relationships between clinical indicators, lesion volumes, diffusion metrics, and SvO_2_ adjusted for age, gender, and TIV in MS patients. False discovery rate (FDR) correction was applied to account for multiple comparisons and correlations. *p* values for group comparisons below two‐sided 0.05 indicated statistical significance.

Regression‐based product‐of‐coefficients mediation analyzes (Baron & Kenny extension; non‐counterfactual framework) were performed using PROCESS v3.2 for SPSS. The simple mediation model (Model 4) examined diffusion metrics in deep brain white matter (WM lesions and NAWM) as mediators of the relationship between SvO_2_ (exposure) and lesion volume/clinical indicators (outcomes). Models adjusted for age, sex, TIV, and disease duration as covariates. Bootstrap sampling (default: 5000 resamples) generated 95% confidence intervals for indirect effects.

## 3 Results

### 3.1 Demographic and Clinical Characteristics

Demographic and clinical characteristics of the participants are summarized in Table 1. The MS cohort exhibited a median Expanded Disability Status Scale (EDSS) score of 2.5 (IQR: 1.0–3.5), indicating mild to moderate disability. The median disease duration was 15 months (IQR: 3–41 months), and the majority of patients (78%) were positive for cerebrospinal fluid oligoclonal bands (OCB). Patients with MS demonstrated significantly poorer cognitive performance compared to HCs, reflected by lower scores on MOCA (26.80 ± 3.11 vs. 28.43 ± 2.12, *p* = 0.003), SDMT (48.57 ± 13.92 vs. 56.37 ± 14.43, *p* = 0.021), CVLT‐II (88.65 ± 26.96 vs. 105.78 ± 25.50, *p* = 0.007), and BVMT‐R (44.10 ± 13.39 vs. 50.89 ± 7.64, *p* = 0.004).

**TABLE 1:** Main demographic, clinical and diffusion MRI features of patients with MS and HCs.

Characteristics	MS (*n* = 79)	HC (*n* = 44)	Test statistic	*p*
Female, *n* (%)	55 (70)	26 (66)	1.393	0.238
Median age (IOR) (years)	33 (29–43)	35 (25–51)	−0.45	0.65
Median disease duration (IQR) (months)	15 (3–41)	—		
Median EDSS (IQR)	2.5 (1–3.5)	—		
OCB Positive (%)	62 (78)	—		
Hct	0.38 (0.04)	0.43 (0.03)	−6.68	< 0.001^a^
MMSE	28.69 (1.87)	29.25 (1.08)	−1.79	0.077
MOCA	26.80 (3.11)	28.43 (2.12)	−3.01	0.003^a^
COWAT	15.28 (6.05)	19.18 (9.26)	−1.99	0.053
SDMT	48.57 (13.92)	56.37 (14.43)	−2.36	0.021^a^
CVLT‐II	88.65 (26.96)	105.78 (25.50)	−2.77	0.007^a^
BVMT‐R	44.10 (13.39)	50.89 (7.64)	−2.95	0.004^a^
Lesion volume (mL)	5.58 (2.21–14.32)			
TIV (mL)	1460 (127)	1480 (149)	−0.792	0.42
Deep brain WM				
FA	0.34 (0.03)	0.36 (0.02)	−3.46	0.001^a^
MD (10^−3^ mm^2^/s)	0.95 (0.10)	0.90 (0.04)	3.71	< 0.001^a^
ODI	0.28 (0.01)	0.27 (0.01)	2.19	0.03^a^
ICVF	0.54 (0.05)	0.58 (0.02)	−5.90	< 0.001^a^

*Note:* Statistical significance with two‐sided *p* < 0.05.

Abbreviations: BVMT‐R, Brief Visuospatial Memory Test‐Revised; COWAT, Controlled Oral Word Association Test; CVLT‐II, Californian Verbal Learning Test II; EDSS, expanded disability status scale; FA, fractional anisotropy; HCT, Hematocrit; ICVF, intracellular volume fraction; IQR, interquartile range; MD, mean diffusivity; MMSE, Mini‐Mental State Examination; MOCA, Montreal Cognitive Assessment; OCB, Oligoclonal bands; ODI, orientation dispersion index; SD, standard deviation; SDMT, Symbol Digit Modality Test.

^a^ Indicated a statistically significant difference compared with HC.

### 3.2 Venous Oxygen Saturation Assessment

After adjusting for cerebral veins pixel, age, gender, and TIV, the venous susceptibility of the ICV in MS patients was higher than HCs (156.10 ± 13.39 vs. 149.34 ± 12.78, *p* = 0.019). Patients with MS exhibited a significantly lower cerebral SvO_2_ compared to HCs (Table 2).

**TABLE 2:** Cerebral venous susceptibility and SvO_2_ at deep cerebral veins of patients with MS and HCs.

Characteristics	MS (*n* = 79)	HC (*n* = 44)	*F* value	*p*
Venous susceptibility (ppb)				
ASV	107.01 (8.81)	109.16 (9.68)	6.52	0.012^a^
TSV	117.76 (10.59)	114.07 (8.38)	0.73	0.40
MLV	108.21 (8.92)	107.63 (9.30)	3.56	0.062
ICV	156.10 (13.39)	149.34 (12.78)	5.70	0.019^a^
SvO_2_ (%)				
ASV	80.44 (1.14)	81.58 (0.65)	27.22	< 0.001^a^
TSV	79.55 (1.26)	81.19 (0.59)	51.63	< 0.001^a^
MLV	80.29 (1.25)	81.61 (0.65)	38.97	< 0.001^a^
ICV	76.56 (1.34)	78.80 (0.86)	55.82	< 0.001^a^

*Note:* Statistical significance with two‐sided *p* < 0.05.

Abbreviations: ASV, anterior septalvein; ICV, internal cerebral vein; MLV, medial lateral ventricle vein; SvO_2_, cerebral venous oxygen saturation; TIV, total intracranial volume; TSV, thalamostriate vein.

^a^ Indicated a statistically significant difference compared with HC.

### 3.3 White Matter Microstructural Assessment

**FIGURE 3:**
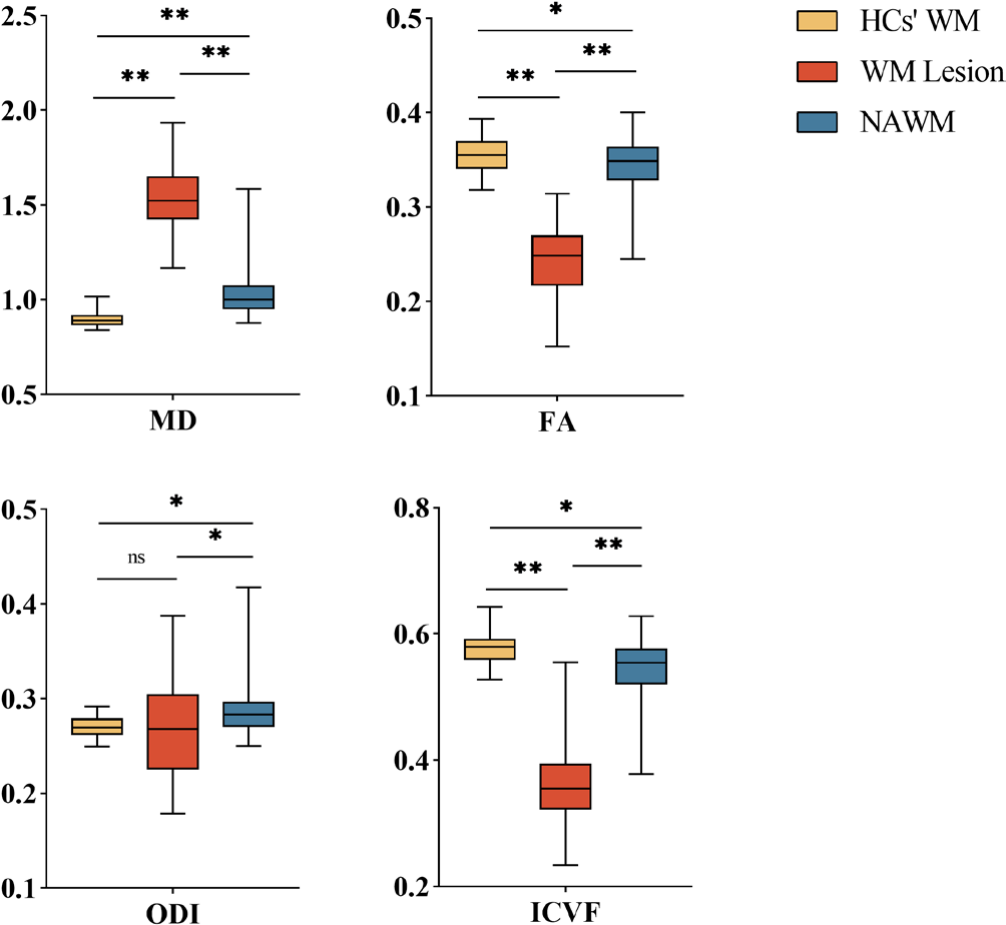
Diffusion tensor‐derived and NODDI measures among HCs' WM (yellow), WM lesion (red) and NAWM (blue). See text for further details. HC, healthy control; NAWM, normal‐appearing white matter; FA, fractional anisotropy; MD, mean diffusivity; ODI, orientation dispersion index; ICVF, intracellular volume fraction; **p < 0.001; *p < 0.05; ns = not significant.

Compared with the deep cerebral WM of HCs, MS patients showed significant microstructural alterations (Figure 3). Lesions exhibited increased MD (1.53 ± 0.17 vs. 0.90 ± 0.04 *p* < 0.001) and reduced FA (0.24 ± 0.04 vs. 0.36 ± 0.02 *p* < 0.001) and ICVF (0.36 ± 0.05 vs. 0.58 ± 0.02 *p* < 0.001). Additionally, NAWM demonstrated increased MD (1.03 ± 0.12 vs. 0.90 ± 0.04 *p* < 0.001) and ODI (0.29 ± 0.03 vs. 0.27 ± 0.01 *p* = 0.006), as well as reduced FA (0.34 ± 0.03 vs. 0.36 ± 0.02 *p* = 0.024) and ICVF (0.54 ± 0.04 vs. 0.58 ± 0.02 *p* = 0.001).

### 3.4 Correlation Between MRI Measures and Clinical Indicators

Partial correlation analyzes indicated that microstructural alteration in NAWM (increased MD and ODI) was significantly correlated with decreased SvO_2_ in the ICV among MS patients (MD, *r* = −0.307, *p* = 0.036; ODI, *r* = −0.279, *p* = 0.036). Strong correlations were found between the microstructure of NAWM and lesion volume (*r* = 0.6–0.8, *p* < 0.001). To exclude the potential influence of individual outliers on the correlation results, we excluded nine outliers (lesion volume > 20 mL, *n* = 9) from the analysis and found that the correlation remained significant (*r* = 0.4–0.6, *p* < 0.001).

**FIGURE 4:**
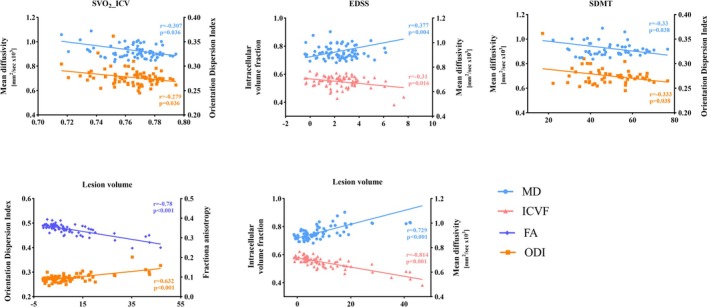
Partial correlation analysis between NAWM FA (purple), MD (blue) or ODI (orange) or ICVF (pink) and clinical and lesion volume adjusted for age, gender, TIV. Continuous line: Statistically significant correlations; see text for further details. FA, fractional anisotropy; MD, mean diffusivity; ICVF, intracellular volume fraction; ODI, Orientation Dispersion Index; EDSS, Expanded Disability Status Scale; SDMT Symbol Digit Modality Test; NAWM, normal‐appearing white matter.

Additionally, in patients with MS, EDSS was positively associated with MD of NAWM (*r* = 0.377, *p* = 0.004) and negatively associated with ICVF of NAWM (*r* = −0.31, *p* = 0.016); SDMT was negatively associated with MD (*r* = −0.33, *p* = 0.038) and ODI (*r* = −0.333, *p* = 0.038) of NAWM (Figure 4). No significant correlations were found between rSvO_2_ and lesion microstructure or lesion volumes.

### 3.5 Mediation Analysis Among SvO_2_ of Deep Vein, Lesion Volumes and NAWM Microstructure Changes

**FIGURE 5:**
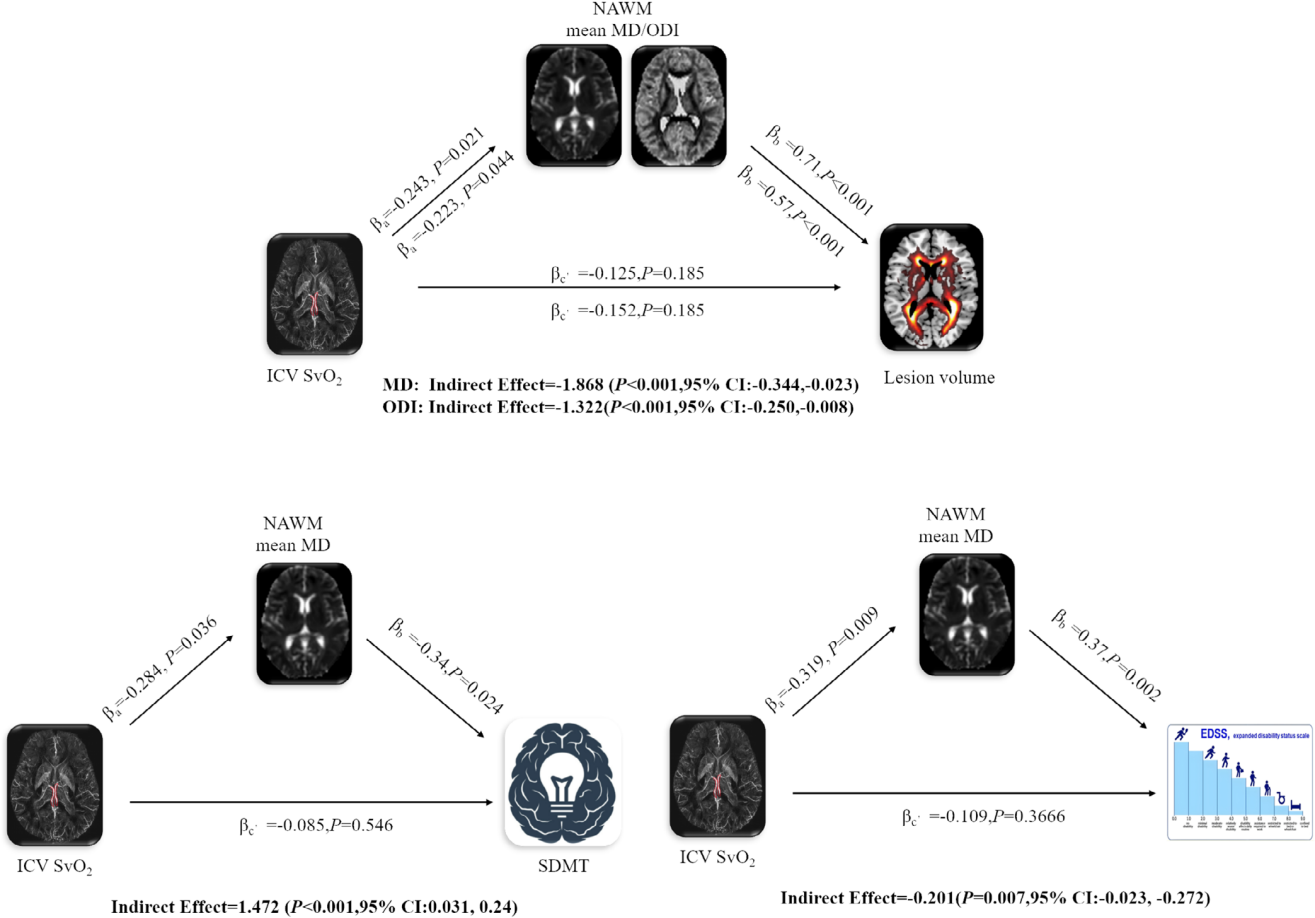
Mediation analysis. The SvO_2_ of ICV and NAWM mean MD/ODI were entered as a predictor and mediator, respectively. Mediation analysis was performed while controlling for the effects of age, sex, disease duration, and TIV. BootCI, bootstrapping Confidence Interval; MD, mean diffusivity; ODI, Orientation Dispersion Index; ICV, internal cerebral vein.

In the MS group, mediation analysis showed that SvO_2_ of ICV indirectly affected WM lesion volumes by damaging NAWM microstructures (Figure 5A). We found NAWM mean MD/ODI had a significant mediating effect on the correlation between SvO_2_ of ICV and lesion volume (from SvO_2_ of ICV to NAWM mean MD/ODI, *β*_*a*_ = −0.243, *p* = 0.021/*β*_*a*_ = −0.223, *p* = 0.044, from NAWM mean MD/ODI to lesion volume, *β*_*b*_ = 0.71, *p* < 0.001/*β*_*b*_ = 0.57, *p* < 0.001). In the patient group, mediation analysis showed that SvO_2_ of ICV indirectly affected EDSS and SDMT by damaging NAWM microstructures (Figure 5B,C). We found NAWM mean MD had a significant mediating effect on the correlation between SvO_2_ of ICV and EDSS (from SvO_2_ of ICV to NAWM mean MD, *β*_*a*_ = −0.319, *p* = 0.009, from NAWM mean MD to EDSS, *β*_*b*_ = 0.37, *p* = 0.002). And NAWM mean MD had a significant mediating effect on the correlation between SvO_2_ of ICV and SDMT (from SvO_2_ of ICV to NAWM mean MD, *β*_*a*_ = −0.284, *p* = 0.036, from NAWM mean MD to SDMT, *β*_*b*_ = −0.34, *p* = 0.024).

However, chain mediation among SvO_2_, white matter microstructural damage, lesion volumes, EDSS, or SDMT is not established. SvO_2_ of the ICV cannot serve as a mediating variable to affect WM microstructural damage and lesion volumes or clinical indicators.

Combining CIS and RRMS patients in the same cohort could introduce heterogeneity due to differences in disease stage, lesion burden, and treatment status. We have performed a supplementary analysis exclusively on the RRMS subgroup (*n* = 66). We are pleased to report that the key findings from this RRMS‐only analysis are highly consistent with those from the combined cohort (see Supporting Information).

## 4 Discussion

Using QSM and multi‐shell diffusion MRI, this study investigates the cross‐sectional associations of cerebral hypoxia with lesion burden and clinical disability (quantified by EDSS and cognitive scores) in MS patients. Three vital findings were found as follows: firstly, significant reduction of SvO_2_ within deep cerebral veins concomitant with microstructural integrity loss in deep brain WM. Secondly, SvO_2_ of the ICV specifically correlated with microstructural damage in NAWM. Additionally, our mediation models suggest that SvO_2_ of the ICV is associated with deep brain WM lesion volume, EDSS, and cognitive function indirectly, via its association with NAWM microstructure in MS patients, although the cross‐sectional design precludes causal inference.

We observed significantly reduced regional SvO_2_ in deep cerebral veins draining deep brain WM territories in MS patients compared to HCs, indicating altered deoxyhemoglobin accumulation in deep cerebral veins. Compared with previous studies, the slightly higher venous oxygen saturation obtained in this work can be attributed to a lowered background threshold. This reduced threshold allows the target vein to be more completely encompassed during segmentation, minimizing signal loss and yielding measurements that more closely reflect the true oxygen saturation. Several factors may contribute to the decreased cerebral rSvO_2_ in MS patients. First, broad consensus has arisen that there is a widespread reduction in cerebral blood flow in MS patients [21], decreased perfusion has been reported in both the corpus callosum [22], NAWM and deep gray matter [23] in CIS, suggesting that reduced perfusion is a primary event in disease development, rather than a secondary response to reduced tissue demand. Hypoperfusion in chronic MS lesions and NAWM may reduce oxygen delivery to cerebral tissues. Second, histopathological studies demonstrate that venous collagenosis induces medullary vein wall thickening, luminal narrowing, and eventually vessel occlusion [24], leading to a prolonged retention time of blood in the capillary bed, and tissue cells continue to uptake oxygen from the blood; also, the increases venous pressure leading to blood diversion to larger veins, causing blood stasis and local accumulation of deoxyhemoglobin. Third, studies of cerebral MS diseases demonstrated that endothelial cell activation occurs before demyelination, suggesting that vascular inflammation may be an early trigger of MS by reducing CBF, further resulting in hypoxia [25]. Fourth, vitamin B12 deficiency and pernicious anemia have been associated with MS, which may reduce the ability to deliver oxygen [26].

However, Sawan et al. [27] reported decreased venous susceptibility and increased SvO_2_ in ICV of MS patients than HCs, which differs from our findings. The reasons for the different findings between our studies might be that almost all MS patients had variable degrees of anemia, which caused the lower Hct levels in the patients, and Hct affects the evaluation of SvO_2_. Although the previous study showed an association between anemia and MS, the study showed that the prevalence of anemia in MS patients (18.7%) is significantly higher than in age‐ and sex‐matched controls without MS (9.5%), and some papers indicate a high rate (39%) of iron deficiency anemia, Sawan et al. did not consider the effect of Hct value when evaluating SvO_2_ in MS patients. In our study, we measured the Hct value in each patient and calculated the SvO_2_ in each patient, reflecting the patients' real oxygen status. In addition, Sawan et al.'s study assumed a constant CBF in these subjects. However, numerous studies have demonstrated a general reduction in perfusion in chronic MS lesions and NAWM [28, 29, 30]. The reduced perfusion will cause an increased oxygen extraction fraction and decreased SvO_2_. Another study found that different lesion types in RRMS have varying susceptibility and OEF values [31], the lesion types of patients in Sawan et al.'s study may differ from our patients.

WM damage is common in MS patients. Consistently with previous findings [12], WM lesion showed higher MD and lower FA and ICVF, NAWM showed higher MD and ODI and lower FA and ICVF compared with HCs. Increased MD indicates microstructural changes that promote free diffusion, such as axonal loss, myelin damage, or edema. Decreased FA occurs when water diffusion becomes less anisotropic, resulting from demyelination, accumulation of inflammatory cells, or axonal damage and ICVF is correlated with neurite density [18], these results confirm that there is active demyelination and inflammation in both lesions and NAWM, as well as loss in neurites, and that structural damage is not confined to focal lesions, the degree of NAWM damage lies in an intermediate range between that of lesions in MS and normal white matter in HCs, representing a transitional zone. This intermediate state reflects the progressive nature of pathological changes in NAWM, which may exhibit subtle structural or functional abnormalities despite appearing normal on conventional imaging. In this study, it was found that ODI did not show significant changes in the lesion, but was elevated in NAWM. Since ODI is a parameter that represents the orientation dispersion of neurites, the lesion may be in a mixed phase of active inflammation which leading to increased ODI and chronic inflammation which leading to decreased ODI, with overall offsetting changes. ODI can indeed increase in WM when highly parallel fibers are disrupted, i.e., structural changes to fibers in NAWM can increase orientation dispersion and reflect tissue damage [32], so the abnormality in ODI may not be related to loss of neurites but could be due to a loss of neurite integrity in NAWM.

Moreover, we found the significant correlation between microstructural damage of NAWM and WM lesion volume. This finding suggests that the NAWM is not merely a passive bystander but is intrinsically linked to the focal lesion burden, potentially serving as a pathological precursor or a contributor to disease progression. The mechanisms underlying NAWM damage are considered multifactorial. One established pathway is retrograde (Wallerian) degeneration, prior studies on the corpus callosum and optic tract have demonstrated a close correlation between DTI metrics and the extent of lesions in connected brain regions and optic nerve, it supports the concept that remote focal lesions lead to axonal transection and trigger Wallerian degeneration [33, 34]. This is corroborated by immunohistochemically evidence demonstrating that active demyelination in focal lesions directly associates with axonal degeneration in adjacent perilesional white matter [35]. However, some studies demonstrated NAWM damage quantified using DTI correlates only partly with the extent of focal lesions and the severity of intrinsic lesion damage [36, 37, 38], which suggests that diffusivity changes in normal‐appearing tissues are not entirely dependent on retrograde degeneration of axons that are transected in focal lesions.

Previous studies about NAWM in MS have demonstrated hypoperfusion and breakdown of blood–brain barrier (BBB) in this area [39]. Investigations specifically targeting the corpus callosum in RRMS have identified correlations between reduced perfusion and decreased mean diffusivity—a hemodynamic profile more indicative of primary ischemic mechanisms than of secondary hypoperfusion [22]. Furthermore, high‐resolution MRI shows abnormal NAWM diffusion metrics such as MD at the edge of the lesion before lesion enlargement [40]. Recent genetic studies have further supported that NAWM microstructural damage is associated with WM lesion progression [41]. Therefore, we posit a dual pathogenesis model for NAWM abnormalities: (1) secondary retrograde degeneration from connected focal lesions, and (2) independent, widespread pathophysiological processes, potentially involving microvascular dysfunction or diffuse inflammation. The correlation we identified between NAWM integrity and total lesion burden underscores their interplay. We hypothesize that the cumulative failure of the NAWM's intrinsic resilience against these insults ultimately culminates in the formation of new lesions.

Furthermore, we found significant correlations between NAWM microstructural damage (MD, ODI, and ICVF) and clinical indicators (EDSS and SDMT scores). In this study, EDSS was utilized as a robust clinical benchmark for gross ambulatory disability and long‐term progression. While it should not be interpreted as a sensitive or comprehensive measure of overall MS disease burden, it provides a standardized metric for physical disability. Meanwhile, SDMT is a sensitive measure of cognitive impairment, particularly information processing speed. A systematic review of Quantitative magnetization transfer imaging in RRMS shown there is a significant negative correlation between the MTR and EDSS scores of NAWM, indicating that microstructural changes in NAWM may contribute to clinical disability [42]. Storelli et al. reported positive correlations between SDMT and FA, and negative correlation with MD in NAWM [43]. Diffuse microstructural injury in NAWM may also impair functional connectivity among cognitively relevant gray matter (GM) regions, contributing to cognitive dysfunction, described as a “disconnection syndrome” [44]. The absence of a significant correlation between WM lesion damage and clinical indicators in our research may be attributed to several factors. MS lesions are highly heterogeneous in their pathological features, this heterogeneity may make it difficult to establish a consistent relationship between specific microstructural changes and clinical outcomes [45]. Also the focal lesions may be compensated by redundant neural networks, particularly if located in non‐critical regions, minimizing their direct clinical impact. NAWM microdamage causes network‐wide inefficiency, whereas focal lesions may spare critical cognitive hubs. In chronic MS, neurodegeneration which driven by NAWM pathology becomes the primary driver of disability, whereas acute lesion effects diminish.

Although we did not find a direct correlation between hypoxia, lesion volume, and clinical indicators, the mediation analysis showed that SvO_2_ of ICV indirectly influenced WM lesion volumes and EDSS/SDMT by inducing microstructure injury in NAWM of MS patients. However, the chain mediation among SvO_2_, WM microstructural damage, lesion volumes and clinical indicators is not established because there is no correlation between lesion volume and clinical indicators. The correlation between WM lesion volumes and EDSS is poor, leading to the “clinico‐radiological paradox” [46]. This lack of correlation is mainly attributed to the presence of additional microscopic abnormalities in the WM and GM, and the assessment of focal WM lesion burden captures only a limited part of the pathological processes affecting the MS brain [47]. MS lesions are the result of both hypoxia and inflammation, and studies have shown that hypoxia can exacerbate inflammation. Hypoxia in WM leads to WM microstructural damage, but appeared normal in MRI; in areas with severe hypoxia, such as the cerebral watershed regions, damage may be further exacerbated by the interaction between hypoxia and inflammation, leading to lesion formation and expansion. The injuries will also have an impact on the patient's physical and cognitive function. Therefore, the SvO_2_ of ICV has the potential to serve as a specific indicator of hypoxia and disease progression in MS patients.

Although our findings highlight important relationships, this study has several limitations. First, the cross‐sectional nature of our study is a key limitation. Although our mediation analysis identifies associative pathways that are consistent with a hypothetical causal model, it cannot establish temporal precedence or causality. The observed associations require validation in longitudinal studies. This limitation is compounded by the absence of direct neuroinflammatory biomarkers (e.g., CSF GFAP or PET imaging), precluding assessment of inflammation‐hypoxia interactions and leaving potential residual confounding unaddressed. Second, we selected the whole segment of deep cerebral veins; although we used a threshold to avoid background tissue, a partial volume effect still exists. Despite the use of a high‐resolution (0.75 mm isotropic) QSM protocol, the accuracy of susceptibility measurements, particularly for smaller cerebral veins, may still be limited by partial volume effects. While this resolution is sufficient for reliably assessing larger intracranial veins such as the internal cerebral veins, it remains challenging to fully resolve smaller structures. Consequently, susceptibility values reported for these smaller veins should be interpreted as estimates, as they are likely underestimated due to signal averaging with the surrounding diamagnetic parenchyma. Third, given the substantial inter‐individual heterogeneity within the patient cohort, which has led to increased variability in disease‐related parameters, the next step involves stratifying patients based on key differentiating features to dynamically characterize the trajectory of blood oxygen changes across different disease stages, thereby refining and strengthening the conclusions of this study. Fourth, in the primary analyzes, CIS and RRMS patients were pooled to increase statistical power and are expected to reflect the disease spectrum from early to established MS. Given the limited sample size of the CIS subgroup and the potential mechanistic differences between CIS and RRMS, this approach constrains our ability to draw definitive conclusions regarding disease dynamics across the full continuum. However, sensitivity analyzes restricted to RRMS patients yielded highly consistent results (see Supporting Information), suggesting that the main findings are not driven by CIS cases. Future studies should expand the CIS cohort to systematically compare CIS and RRMS, thereby further elucidating the similarities and distinctions in their disease progression.

In MS patients, lower cerebral SvO_2_ (compared with HCs) is statistically associated with microstructural alterations in the NAWM. Our mediation models are consistent with a pathway whereby lower SvO_2_ is associated with greater lesion burden and worse functional scores via its association with NAWM damage. These findings support the exploratory value of SvO_2_ and NAWM integrity as potential biomarkers for monitoring MS progression, which warrants validation in future longitudinal studies.

## Author Contributions

X.W., S.X., C.C., and Y.D. contributed to the conception and design of the manuscript. X.W. and H.W. drafted the original manuscript. X.W. and WHY analyzed and interpreted the patient data. X.W. and K.L. were responsible for reviewing the MRI imaging. X.W. and Z.Z. were responsible for MRI imaging analysis. X.W., Y.D., D.T., and A.G. were responsible for collecting clinical and radiological data. S.X., C.C., and Y.D. supervised and revised the manuscript. All authors read and approved the final manuscript.

## Funding

This work was supported by Natural Scientific Foundation of China (Grant 82171916), Natural Science Foundation of Tianjin (Grants 21CYBJC01580 and 21JCYBJC01060), Tianjin Healthy High‐Level Talent Selection and Training Project (Grant TJSQNYXXR‐D2‐143) and Tianjin Health Research Project (Grant TJWJ2023QN031).

## Conflicts of Interest

The authors declare no conflicts of interest.

## Supporting information


**Data S1:** acn370354‐sup‐0001‐Supinfo.docx.

## Data Availability

Data are available upon reasonable request. The data set used and analyzed during the current study are available from the corresponding author on reasonable request.
