# Supplementary material for: Normal‐Appearing White Matter Injury Mediates Chronic Deep Venous Hypoxia and Disease Progression in Multiple Sclerosis
Source: Ann Clin Transl Neurol. 2026 Feb 27;13(8):1686–96. doi: 10.1002/acn3.70354 (PMC13395016; doi:10.1002/acn3.70354)
Supplement: Supplementary file 1 [file ACN3-13-1686-s001.docx]

**Supplementary Materials**

**Results**

**Demographic and clinical characteristics**

Demographic and clinical characteristics of the participants are summarized in Table 1. Patients with RRMS demonstrated significantly poorer cognitive performance compared to HCs, reflected by lower scores in MOCA (26.91±3.21 vs 28.43±2.12 *P* = 0.008), SDMT (48.30±14.23 vs 56.37±14.43 *P* = 0.023), CVLT-II (90.49±27.61 vs 105.78±25.50 *P* = 0.021), and BVMT-R (44.79±13.00 vs 50.89±7.64 *P* = 0.013).

**Venous oxygen saturation assessment**

After adjusting for cerebral veins pixel, age, gender, and TIV, the venous susceptibility of the ICV in RRMS patients was higher than HCs (157.56±13.59 vs 149.34±12.78 *P*=0.002). Patients with RRMS exhibited a significantly lower cerebral SvO_2_ compared to HCs (76.64±1.82 vs 78.57±0.98 *P*<0.001) (Table S2).

**White Matter Microstructural Assessment**

Compared with the deep cerebral WM of HCs, RRMS patients showed significant microstructural alterations (Figure S1). Lesions exhibited increased MD (1.51±0.15 vs 0.90±0.04 *P*<0.001) and reduced FA (0.25±0.04 vs 0.36±0.02 *P*<0.001) and ICVF (0.36±0.06 vs 0.58±0.02 *P*<0.001). Additionally, NAWM demonstrated increased MD (1.04±0.13 vs 0.90±0.04 *P*<0.001) and ODI (0.29±0.03 vs 0.27±0.01 P=0.011), as well as reduced FA (0.33±0.03 vs 0.36±0.02 *P*<0.001) and ICVF (0.53±0.05 vs 0.58±0.02 *P*<0.001).

**Correlation between MRI Measures and Clinical Indicators**

Partial correlation analyses indicated that microstructural alteration in NAWM (increased MD and ODI) were significantly correlated with decreased SvO_2_ in the ICV among RRMS patients (MD, r=-0.271, *P*=0.04; ODI, r=-0.290, *P*=0.027). Strong correlations were found between the microstructure of NAWM and lesion volume (r=0.7-0.8, *P* <0.001).

Additionally, in patients with RRMS, EDSS was positively associated with MD of NAWM (r=0.327, *P*=0.012) and negatively associated with ICVF of NAWM (r=-0.278, *P*=0.033), SDMT was negatively associated with MD (r=-0.398, *P*=0.011) and ODI (r=-0.334, *P*=0.035) of NAWM (Figure S2). No significant correlations were found between rSvO_2_ and lesion microstructure or lesion volumes.

**Mediation analysis among SvO_2_ of deep vein, lesion volumes and NAWM microstructure changes.**

In the MS group, mediation analysis showed that SvO_2_ of ICV indirectly affected WM lesion volumes by damaging NAWM microstructures (Figure S3A). We found mean MD/ODI of NAWM had a significant mediating effect on the relationship between SvO2 of ICV and lesion volume (from SvO_2_ of ICV to mean MD/ODI of NAWM, βa=-0.233, *P*=0.04/βa=-0.252, P=0.027, from mean MD/ODI to lesion volume, βb =0.76, *P*<0.001/βb =0.72, *P*<0.001). In the patient group, mediation analysis showed that SvO_2_ of ICV indirectly affected EDSS and SDMT by damaging NAWM microstructures (Figure S3B, C). We found mean MD of NAWM had a significant mediating effect on the relationship between SvO_2_ of ICV and EDSS (from SvO_2_ of ICV to mean MD of NAWM, βa=-0.176, *P*=0.042, from mean MD to EDSS, βb =0.37, *P*=0.004). And NAWM mean MD had a significant mediating effect on the relationship between SvO_2_ of ICV and SDMT (from SvO_2_ of ICV to mean MD of NAWM, βa=-0.351, *P*=0.019, from mean MD to SDMT, βb =-0.38, *P*=0.013).

However, chain mediation among SvO_2_, white matter microstructural damage, lesion volumes, EDSS or SDMT is not establish. SvO_2_ of the ICV cannot serve as a mediating variable to affect WM microstructural damage and lesion volumes or clinical indicators.

Table S1: Main demographic, clinical and diffusion MRI features of patients with RRMS and HCs

| Characteristics | | RRMS(n=66) | | HCs(n=44) | Test Statistic | | | *P* |
| --- | --- | --- | --- | --- | --- | --- | --- | --- |
| Female, n(%) | | 44(66) | 26(60) | | | 0.66 | | 0.418 |
| Median age(IOR)(years) | | 31(26-37) | 35(25-51) | | | -1.89 | | 0.06 |
| Median disease  Duration(IQR)(months) | | 19(7-69) | | - | |  | |  |
| Median EDSS (IQR) | | 2.5(1-3.5) | | - | |  | |  |
| OCB Positive(%) | | 57(86) | | - | |  | |  |
| Hct | | 0.38(0.05) | | 0.43(0.03) | | -6.23 | | <0.001^*^ |
| MMSE | | 28.77(1.93) | | 29.25(1.08) | | -1.46 | | 0.146 |
| MOCA | | 26.91(3.21) | | 28.43(2.12) | | -2.70 | | 0.008^*^ |
| COWAT | | 15.77(6.39) | | 19.18(9.26) | | -1.70 | | 0.065 |
| SDMT | 48.30(14.23) | | | 56.37(14.43) | | -2.33 | | 0.023^*^ |
| CVLT-II | 90.49(27.61) | | | 105.78(25.50) | | -2.36 | | 0.021^*^ |
| BVMT-R | 44.79(13.00) | | | 50.89(7.64) | | -2.54 | | 0.013^*^ |
| Lesion volume(mL) | 7.04(3.0-16.29) | | |  | | |  |  |
| TIV(mL) | 1463(130) | | | 1480(149) | -0.65 | | | 0.517 |
| Deep brain WM |  | | |  |  | | |  |
| FA | 0.33(0.03) | | | 0.36(0.02) | -4.09 | | | <0.001^*^ |
| MD(10^−3^ mm^2^/s) | 0.95(0.10) | | | 0.90(0.04) | 3.73 | | | <0.001^*^ |
| ODI | 0.27(0.01) | | | 0.27(0.01) | 2.53 | | | 0.013^*^ |
| ICVF | 0.53(0.05) | | | 0.58(0.02) | -6.59 | | | <0.001^*^ |

Table S2: Cerebral venous susceptibility and SvO_2_ at deep cerebral veins of patients with RRMS and HCs

| Characteristics | RRMS(n=66) | HCs(n=44) | *F* value | *P* |
| --- | --- | --- | --- | --- |
| Venous susceptibility(ppb) |  |  |  |  |
| ASV | 106.57 (8.89) | 109.16(9.68) | 0.064 | 0.38 |
| TSV | 118.89(11.03) | 114.07(8.38) | 6.226 | 0.018^*^ |
| MLV | 108.36(9.18) | 107.63(9.30) | 0.143 | 0.70 |
| ICV | 157.56(13.59) | 149.34(12.78) | 7.885 | 0.002^*^ |
| SvO_2_(%) |  |  |  |  |
| ASV | 80.45(1.20) | 81.42(0.74) | 35.508 | <0.001^*^ |
| TSV | 79.44(1.40) | 81.01(0.80) | 50.774 | <0.001^*^ |
| MLV | 80.27(1.29) | 81.62(1.43) | 26.856 | <0.001^*^ |
| ICV | 76.64(1.82) | 78.57(0.98) | 47.851 | <0.001^*^ |

Figure S1. Diffusion tensor­derived and NODDI measures among HCs’ WM (yellow), WM lesion (red) and NAWM (blue). See text for further details.

HCs, healthy controls; NAWM, normal-­appearing white matter; FA, fractional anisotropy; MD, mean diffusivity; ODI, orientation dispersion index; ICVF, intracellular volume fraction; ***P*<0.001; **P*<0.05; ns = not significant

Figure S2. Partial correlation analysis between NAWM FA (purple), MD (blue) or ODI (orange) or ICVF (pink) and clinical and lesion volume adjusted for age, gender, TIV.

Continuous line: statistically significant correlations; See text for further details.

FA, fractional anisotropy; MD, mean diffusivity; ICVF, intracellular volume fraction; ODI, Orientation Dispersion Index; EDSS, Expanded Disability Status Scale; SDMT Symbol Digit Modality Test; NAWM, normal-­appearing white matter.

Figure S3. Mediation analysis. The SvO_2_ of ICV and mean MD/ODI of NAWM were entered as a predictor and mediator, respectively. Mediation analysis was performed while controlling for the effects of age, sex, disease duration and TIV. BootCI, bootstrapping Confidence Interval; MD, mean diffusivity; ODI, Orientation Dispersion Index; ICV, internal cerebral vein
