# Supplementary material for: Normal‐Appearing White Matter Injury Mediates Chronic Deep Venous Hypoxia and Disease Progression in Multiple Sclerosis
Source: Ann Clin Transl Neurol. 2026 Feb 27:10.1002/acn3.70354. Online ahead of print. doi: 10.1002/acn3.70354 (PMC13395016; doi:10.1002/acn3.70354)
Supplement: Supplementary file 1 — Data S1: acn370354‐sup‐0001‐Supinfo.docx. [file ACN3-9999-0-s001.docx]

**Supplementary Materials**

**Results**

**Demographic and clinical characteristics**

Demographic and clinical characteristics of the participants are summarized in Table 1. Patients with RRMS demonstrated significantly poorer cognitive performance compared to HCs, reflected by lower scores in MOCA (26.91±3.21 vs 28.43±2.12 *P* = 0.008), SDMT (48.30±14.23 vs 56.37±14.43 *P* = 0.023), CVLT-II (90.49±27.61 vs 105.78±25.50 *P* = 0.021), and BVMT-R (44.79±13.00 vs 50.89±7.64 *P* = 0.013).

**Venous oxygen saturation assessment**

After adjusting for cerebral veins pixel, age, gender, and TIV, the venous susceptibility of the ICV in RRMS patients was higher than HCs (157.56±13.59 vs 149.34±12.78 *P*=0.002). Patients with RRMS exhibited a significantly lower cerebral SvO_2_ compared to HCs (76.64±1.82 vs 78.57±0.98 *P*<0.001) (Table S2).

**White Matter Microstructural Assessment**

Compared with the deep cerebral WM of HCs, RRMS patients showed significant microstructural alterations (Figure S1). Lesions exhibited increased MD (1.51±0.15 vs 0.90±0.04 *P*<0.001) and reduced FA (0.25±0.04 vs 0.36±0.02 *P*<0.001) and ICVF (0.36±0.06 vs 0.58±0.02 *P*<0.001). Additionally, NAWM demonstrated increased MD (1.04±0.13 vs 0.90±0.04 *P*<0.001) and ODI (0.29±0.03 vs 0.27±0.01 P=0.011), as well as reduced FA (0.33±0.03 vs 0.36±0.02 *P*<0.001) and ICVF (0.53±0.05 vs 0.58±0.02 *P*<0.001).

**Correlation between MRI Measures and Clinical Indicators**

Partial correlation analyses indicated that microstructural alteration in NAWM (increased MD and ODI) were significantly correlated with decreased SvO_2_ in the ICV among RRMS patients (MD, r=-0.271, *P*=0.04; ODI, r=-0.290, *P*=0.027). Strong correlations were found between the microstructure of NAWM and lesion volume (r=0.7-0.8, *P* <0.001).

Additionally, in patients with RRMS, EDSS was positively associated with MD of NAWM (r=0.327, *P*=0.012) and negatively associated with ICVF of NAWM (r=-0.278, *P*=0.033), SDMT was negatively associated with MD (r=-0.398, *P*=0.011) and ODI (r=-0.334, *P*=0.035) of NAWM (Figure S2). No significant correlations were found between rSvO_2_ and lesion microstructure or lesion volumes.

**Mediation analysis among SvO_2_ of deep vein, lesion volumes and NAWM microstructure changes.**

In the MS group, mediation analysis showed that SvO_2_ of ICV indirectly affected WM lesion volumes by damaging NAWM microstructures (Figure S3A). We found mean MD/ODI of NAWM had a significant mediating effect on the relationship between SvO2 of ICV and lesion volume (from SvO_2_ of ICV to mean MD/ODI of NAWM, βa=-0.233, *P*=0.04/βa=-0.252, P=0.027, from mean MD/ODI to lesion volume, βb =0.76, *P*<0.001/βb =0.72, *P*<0.001). In the patient group, mediation analysis showed that SvO_2_ of ICV indirectly affected EDSS and SDMT by damaging NAWM microstructures (Figure S3B, C). We found mean MD of NAWM had a significant mediating effect on the relationship between SvO_2_ of ICV and EDSS (from SvO_2_ of ICV to mean MD of NAWM, βa=-0.176, *P*=0.042, from mean MD to EDSS, βb =0.37, *P*=0.004). And NAWM mean MD had a significant mediating effect on the relationship between SvO_2_ of ICV and SDMT (from SvO_2_ of ICV to mean MD of NAWM, βa=-0.351, *P*=0.019, from mean MD to SDMT, βb =-0.38, *P*=0.013).

However, chain mediation among SvO_2_, white matter microstructural damage, lesion volumes, EDSS or SDMT is not establish. SvO_2_ of the ICV cannot serve as a mediating variable to affect WM microstructural damage and lesion volumes or clinical indicators.

Table S1: Main demographic, clinical and diffusion MRI features of patients with RRMS and HCs

| Characteristics | | RRMS(n=66) | | HCs(n=44) | Test Statistic | | | *P* |
| --- | --- | --- | --- | --- | --- | --- | --- | --- |
| Female, n(%) | | 44(66) | 26(60) | | | 0.66 | | 0.418 |
| Median age(IOR)(years) | | 31(26-37) | 35(25-51) | | | -1.89 | | 0.06 |
| Median disease  Duration(IQR)(months) | | 19(7-69) | | - | |  | |  |
| Median EDSS (IQR) | | 2.5(1-3.5) | | - | |  | |  |
| OCB Positive(%) | | 57(86) | | - | |  | |  |
| Hct | | 0.38(0.05) | | 0.43(0.03) | | -6.23 | | <0.001^*^ |
| MMSE | | 28.77(1.93) | | 29.25(1.08) | | -1.46 | | 0.146 |
| MOCA | | 26.91(3.21) | | 28.43(2.12) | | -2.70 | | 0.008^*^ |
| COWAT | | 15.77(6.39) | | 19.18(9.26) | | -1.70 | | 0.065 |
| SDMT | 48.30(14.23) | | | 56.37(14.43) | | -2.33 | | 0.023^*^ |
| CVLT-II | 90.49(27.61) | | | 105.78(25.50) | | -2.36 | | 0.021^*^ |
| BVMT-R | 44.79(13.00) | | | 50.89(7.64) | | -2.54 | | 0.013^*^ |
| Lesion volume(mL) | 7.04(3.0-16.29) | | |  | | |  |  |
| TIV(mL) | 1463(130) | | | 1480(149) | -0.65 | | | 0.517 |
| Deep brain WM |  | | |  |  | | |  |
| FA | 0.33(0.03) | | | 0.36(0.02) | -4.09 | | | <0.001^*^ |
| MD(10^−3^ mm^2^/s) | 0.95(0.10) | | | 0.90(0.04) | 3.73 | | | <0.001^*^ |
| ODI | 0.27(0.01) | | | 0.27(0.01) | 2.53 | | | 0.013^*^ |
| ICVF | 0.53(0.05) | | | 0.58(0.02) | -6.59 | | | <0.001^*^ |

Table S2: Cerebral venous susceptibility and SvO_2_ at deep cerebral veins of patients with RRMS and HCs

| Characteristics | RRMS(n=66) | HCs(n=44) | *F* value | *P* |
| --- | --- | --- | --- | --- |
| Venous susceptibility(ppb) |  |  |  |  |
| ASV | 106.57 (8.89) | 109.16(9.68) | 0.064 | 0.38 |
| TSV | 118.89(11.03) | 114.07(8.38) | 6.226 | 0.018^*^ |
| MLV | 108.36(9.18) | 107.63(9.30) | 0.143 | 0.70 |
| ICV | 157.56(13.59) | 149.34(12.78) | 7.885 | 0.002^*^ |
| SvO_2_(%) |  |  |  |  |
| ASV | 80.45(1.20) | 81.42(0.74) | 35.508 | <0.001^*^ |
| TSV | 79.44(1.40) | 81.01(0.80) | 50.774 | <0.001^*^ |
| MLV | 80.27(1.29) | 81.62(1.43) | 26.856 | <0.001^*^ |
| ICV | 76.64(1.82) | 78.57(0.98) | 47.851 | <0.001^*^ |

**
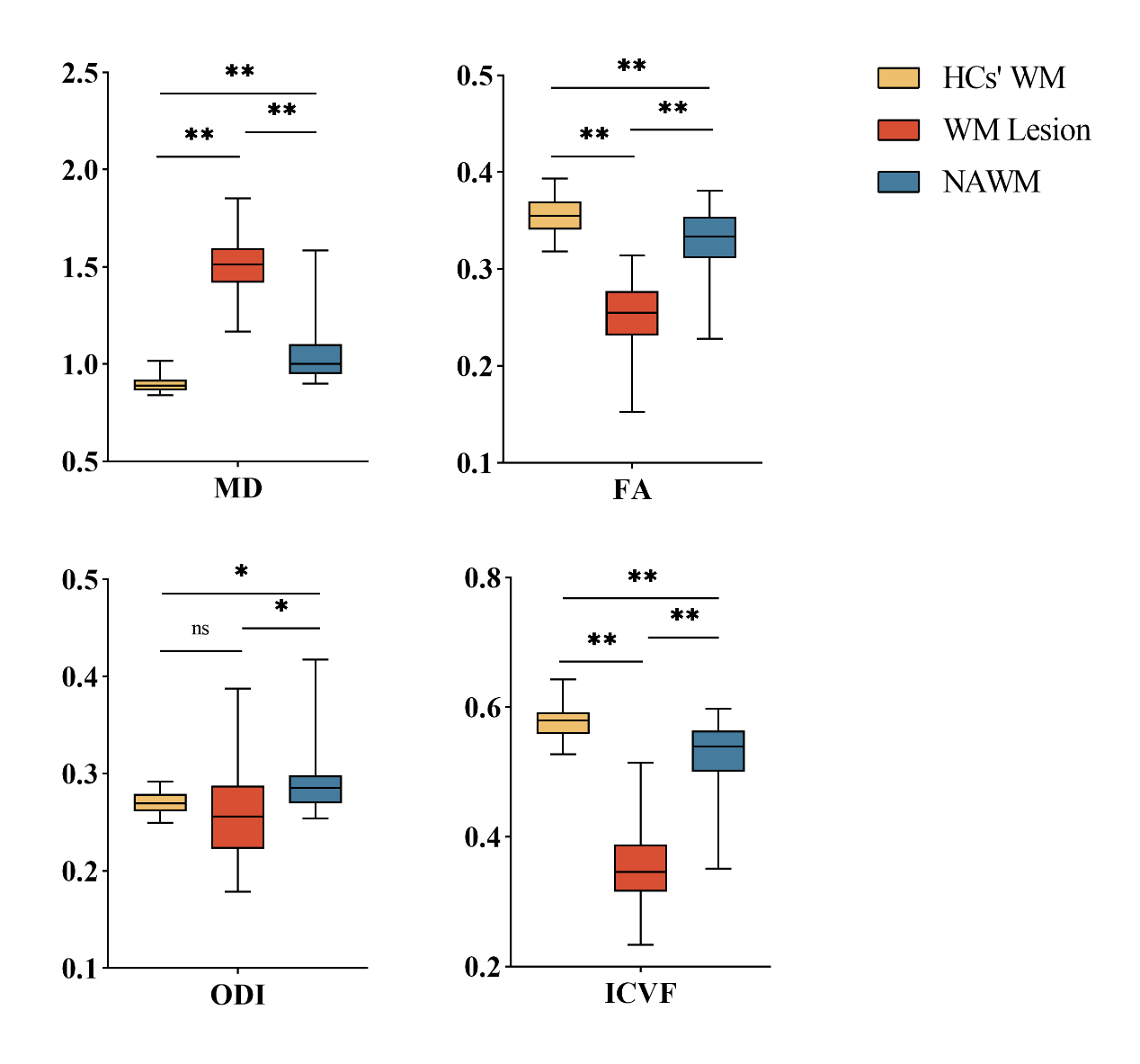
**

Figure S1. Diffusion tensor­derived and NODDI measures among HCs’ WM (yellow), WM lesion (red) and NAWM (blue). See text for further details.

HCs, healthy controls; NAWM, normal-­appearing white matter; FA, fractional anisotropy; MD, mean diffusivity; ODI, orientation dispersion index; ICVF, intracellular volume fraction; ***P*<0.001; **P*<0.05; ns = not significant

**
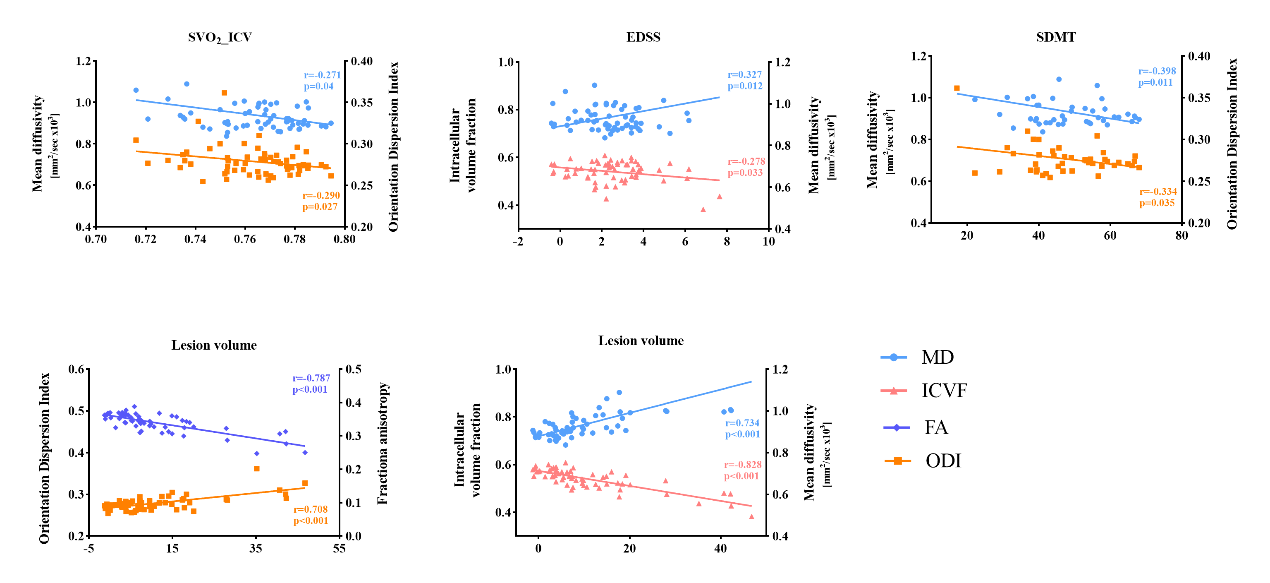
**

Figure S2. Partial correlation analysis between NAWM FA (purple), MD (blue) or ODI (orange) or ICVF (pink) and clinical and lesion volume adjusted for age, gender, TIV.

Continuous line: statistically significant correlations; See text for further details.

FA, fractional anisotropy; MD, mean diffusivity; ICVF, intracellular volume fraction; ODI, Orientation Dispersion Index; EDSS, Expanded Disability Status Scale; SDMT Symbol Digit Modality Test; NAWM, normal-­appearing white matter.

**
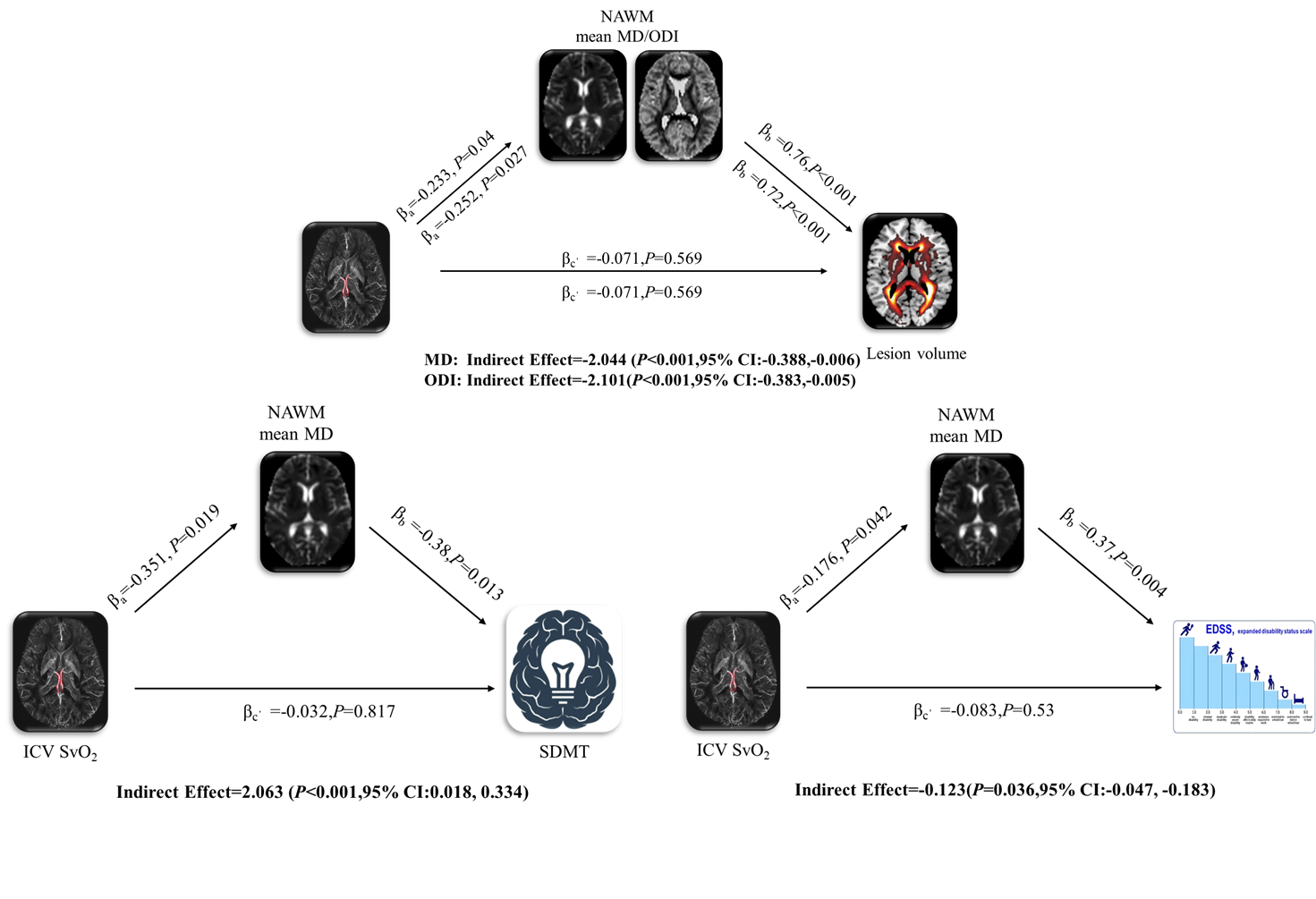
**Figure S3. Mediation analysis. The SvO_2_ of ICV and mean MD/ODI of NAWM were entered as a predictor and mediator, respectively. Mediation analysis was performed while controlling for the effects of age, sex, disease duration and TIV. BootCI, bootstrapping Confidence Interval; MD, mean diffusivity; ODI, Orientation Dispersion Index; ICV, internal cerebral vein
